# The role of nucleation in sickle cell pathophysiology: opportunities for innovative treatments

**DOI:** 10.1097/MS9.0000000000002705

**Published:** 2024-11-04

**Authors:** Emmanuel Ifeanyi Obeagu, Callistus A. Akinleye, Getrude Uzoma Obeagu

**Affiliations:** a Department of Biomedical and Laboratory Science, Africa University, Zimbabwe; b Department of Community Medicine, Osun State University, Osogbo, Nigeria; c School of Nursing Science, Kampala International University, Ishaka, Uganda

**Keywords:** hemoglobin polymerization, nucleation, pathophysiology, sickle cell anemia, therapeutic strategies

## Abstract

Sickle cell anemia (SCA) is driven by the polymerization of hemoglobin S (HbS), where the nucleation process plays a central role in initiating sickling episodes. Advances in structural biology and computational modeling have significantly deepened our understanding of this process. High-resolution crystallography has elucidated the structural changes in deoxygenated HbS that promote nucleation, revealing critical interactions between valine-substituted β-globin chains. Cryo-electron microscopy (cryo-EM) has provided detailed visualizations of early-stage polymerization, capturing the formation of small HbS aggregates, which are essential for understanding the dynamics of nucleation in physiological conditions. Additionally, computational modeling has offered valuable insights into the kinetics of HbS nucleation, enabling the prediction of polymer formation under varying oxygen tensions. Molecular dynamics simulations have been instrumental in identifying key factors that modulate nucleation, such as intracellular HbS concentration, pH, and ionic strength. These simulations also suggest that heterogeneous nucleation, facilitated by cellular surfaces or macromolecules, may accelerate the sickling process, highlighting potential therapeutic targets for disrupting this interaction. Together, these techniques have led to new opportunities for innovative treatments. For instance, voxelotor, a drug developed using structural insights, binds to HbS and prevents its deoxygenation, reducing nucleation rates. Other strategies, such as CRISPRbased gene editing and allosteric modulators, are emerging as potential therapeutic avenues for altering nucleation kinetics, offering hope for more effective treatments to mitigate the clinical severity of SCA.

## Introduction

Sickle cell anemia (SCA), the most severe form of sickle cell disease(SCD), isa genetically inherited disorder characterized bythe presence of abnormal hemoglobin (HbS) in red blood cells (RBCs). Due to a single-point mutation in the β-globin gene, HbS polymerizes under deoxygenated conditions, causing RBCs to deform into a sickle shape. These sickled cells lead to various clinical complications, such as vaso-occlusion, hemolysis, and organ damage. Globally, SCA is one of the most prevalent genetic disorders, disproportionately affecting populations of African descent and other regions where malaria was historically endemic. The burden of SCA is significant, and it remains a major public health issue in many low-income countries and middleincome countries (LMICs)^[1-4]^. SCA is most prevalent in sub-Saharan Africa, where ~ 80% of the world’s sickle cell births occur annually. The WHO estimates that more than 300 000 infants are born with SCA each year globally, and around 230 000 of these births are in Africa. Nigeria alone accounts for over 100 000 SCA births annually, representing the largest population of individuals with SCD worldwide. The prevalence of the HbS gene mutation in West Africa, particularly in regions such as Ghana, the Democratic Republic of Congo, and Angola, exceeds 20%. This high burden reflects the selective advantage that the HbS trait confers against malaria, resulting in the persistence of the mutation in regions where malaria was historically endemic^[5-7]^. Despite advancements in medical research, SCA remains a significant cause of mortality, particularly in childhood. In sub-Saharan Africa, where access to comprehensive care is limited, over 50% of children born with SCA die before the age of five due to complications such as severe infections, acute chest syndrome, and stroke. In regions where newborn screening and early interventions are scarce, mortality rates are even higher. In contrast, countries with well-established healthcare systems, such as the United States and parts of Europe, have significantly lower mortality rates, with many patients surviving into adulthood. However, morbidity remains high, with frequent hospitalizations, chronic pain, and a reduced quality of life^[8-10]^.

**Highlights**

- Nucleation mechanisms: Understanding how HbS nucleates and polymerizes is crucial for developing targeted therapies.
- Precision medicine: Tailoring treatments to individuals’ genetic profiles promises improved outcomes.
- Gene editing technologies: CRISPR/Cas9 offers the potential for permanent correction of the genetic defect underlying sickle cell anemia.
- Nanomedicine advancements: Nanoparticle drug delivery systems enhance drug efficacy and reduce side effects.
- Combination therapies: Targeting multiple aspects of SCD pathophysiology offers comprehensive treatment approaches with synergistic benefits

Regional variations in the prevalence and clinical outcomes of SCA reflect both genetic factors and differences in healthcare infrastructure. For instance, in sub-Saharan Africa, neonatal screening programs are still underdeveloped, and early interventions, such as prophylactic antibiotics and vaccinations, are not widely accessible. This leads to high childhood mortality rates. In contrast, countries in the Middle East and India, where the HbS gene is also prevalent due to historical malaria exposure, have better-established newborn screening and treatment protocols, resulting in improved survival outcomes. In India, for example, the prevalence of SCA is particularly high among tribal populations, with rates of sickle cell trait ranging from 5 to 40% depending on the region^[11,12]^. The economic and social impact of SCA in LMICs is immense. In many African countries, SCArelated complications account for a significant proportion of hospital admissions and healthcare costs. Families face the dual burden of frequent hospitalizations and long-term disability, which often lead to financial strain and loss of productivity. The chronic nature of the disease, coupled with the lack of specialized care, contributes to ongoing health disparities, perpetuating cycles of poverty and poor health outcomes for affected individuals. Public health initiatives in these regions have yet to fully address the needs of individuals with SCA, and more robust strategies are required to improve diagnosis, treatment, and longterm care^[13,14]^. The burden of SCA is not limited to Africa, as large diaspora populations in the United States, Europe, and the Caribbean also face significant health challenges. In the United States, ~ 100 000 individuals live with SCD, with African Americans comprising more than 90% of the affected population. The prevalence of SCD in African American communities is around 1 in 365 births, with ~1 in 13 African Americans carrying thesicklecell trait.Despitetheavailabilityofadvancedhealthcare services in the US, individuals with SCA experience substantial morbidity and reduced life expectancy compared to the general population. Vaso-occlusive crises (VOCs) remain the leading cause of hospitalization, and complications such as pulmonary hypertension, renal dysfunction, and stroke are common^[15]^.

In Europe, SCA is increasingly recognized as a public health issue due to immigration from regions with a high prevalence of SCD. Countries such as the United Kingdom and France have seen a rise in SCA cases, particularly among individuals of African and Caribbean descent. In the U.K., where SCD is the most common genetic disorder, ~ 15 000 individuals live with the disease. Newborn screening programs and comprehensive care protocols have improved survival rates, but individuals with SCA continue to face significant healthcare challenges, including chronic pain, frequent hospitalizations, and complications such as organ damage^[16]^. SCA is also prevalent in the Caribbean, where populations with African ancestry are disproportionately affected. In countries such as Jamaica, SCA is a significant contributor to childhood morbidity and mortality. The Jamaican cohort study, which began in the 1970s, has provided valuable insights into the natural history of SCA and the benefits of early intervention. Despite these advances, resource limitations continue to hinder the widespread implementation of effective screening and treatment programs across the region. Access to comprehensive care, including hydroxyurea therapy and regular monitoring for complications, remains limited for many individuals^[17]^. Addressing the global burden of SCA requires a multifaceted approach that includes improvements in healthcare infrastructure, expanded newborn screening programs, and increased access to affordable treatments. In high-prevalence regions, such as sub-Saharan Africa, scaling up early diagnosis and preventive care is critical for reducing childhood mortality. International collaborations and partnerships, such as the Global Sickle Cell Disease Network, are essential for building capacity and sharing best practices for managing the disease. The development of cost-effective treatments, such as gene therapy, also holds promise for reducing the global burden of SCA^[18]^.

This paper aims to comprehensively explore the current state of knowledge regarding nucleation mechanisms in SCA and their implications for disease management.

## Nucleation mechanisms in sickle cell pathophysiology

Nucleation plays a pivotal role in the pathophysiology of sickle cell anemia (SCA), as it marks the initiation of hemoglobin S (HbS) polymerization, leading to the sickling of red blood cells (RBCs). Nucleation occurs when deoxygenated HbS molecules aggregate into long, stiff polymers within RBCs, deforming them into a characteristic sickle shape. These sickled cells are less flexible and more prone to causing vaso-occlusive episodes (VOCs), one of the hallmark complications of SCA. The process of nucleation is influenced by several factors, including HbS concentration, pH levels, oxygen tension, and the presence of modifiers like 2,3-diphosphoglycerate (2,3-DPG) and inositol hexaphosphate (IHP). Adetailed understanding ofthese factorsis critical for linking nucleation mechanisms to clinical manifestations and identifying potential therapeutic targets^[19]^.

## Hemoglobin S concentration

The concentration of HbS within RBCs is a major determinant of nucleation rate. Higher intracellular HbS concentrations increase the likelihood of polymer formation due to the elevated probability of HbS molecules coming into contact and aggregating. Quantitatively, it has been observed that nucleation rates are exponentially dependent on HbS concentration. For instance, at an HbS concentration of ~20 g/dl, nucleation occurs within a few seconds under hypoxic conditions, leading to rapid sickling. However, when HbS concentrations fall below 15 g/dl, nucleation rates decrease significantly, delaying polymerization and reducing the severity of sickling episodes. This relationship between HbS concentration and nucleation rate underscores the importance of therapeutic strategies aimed at reducing intracellular HbS levels, such as hydroxyurea treatment, which increases the production of fetal hemoglobin (HbF) that interferes with HbS polymerization^[20]^.

pH and nucleation kinetics pH is another critical factor that modulates HbS nucleation. Acidic conditions (low pH) promote polymerization by enhancing the hydrophobic interactions between deoxygenated HbS molecules. A pH below 7.4 accelerates the formation of HbS polymers, increasing nucleation rates and thereby exacerbating sickling under hypoxic conditions. Studies have shown that lowering the pH from 7.4 to 7.0 can increase nucleation rates by more than 50%, making acidosis a significant contributor to VOCs. In clinical practice, metabolic acidosis, which can result from dehydration, infection, or strenuous exercise, is often observed in patients with SCA and is associated with an increased frequency of sickle cell crises. Correcting acidosis in thesepatients is crucial for managing their condition and preventing acute sickling episodes.2^[1]^

## Oxygen tension

Oxygen tension directly affects the polymerization of HbS because the deoxygenated form of HbS is the primary trigger for nucleation. When oxygen tension drops, such as during hypoxic events or physical exertion, HbS deoxygenates and becomes prone to polymerization. The rate of nucleation in RBCs is highly sensitive to changes in oxygen tension, with nucleation rates increasing dramatically as oxygen levels fall below 40 mmHg. In severe hypoxia (e.g. oxygen tension below 20 mmHg), nucleation can occur almost instantaneously, leading to rapid sickling and subsequent vaso-occlusion. This explains why patients with SCA often experience acute crises in response to environmental or physiological conditions that induce hypoxia, such as high altitudes, infections, or respiratory conditions. Oxygen supplementation is a common clinical intervention to prevent or mitigate the effects of hypoxia-induced sickling^[21]^.

## Role of 2,3-diphosphoglycerate (2,3-DPG)

2,3-DPG is a metabolic intermediate that binds to deoxygenated hemoglobin, stabilizing its T-state and facilitating the release of oxygen from hemoglobin to tissues. In individuals with SCA, 2,3DPG levels are often elevated, whichpromotes the deoxygenation of HbS and accelerates nucleation. Higher levels of 2,3-DPG reduce the affinity of hemoglobin for oxygen, lowering oxygen saturation in RBCs, and increasing the likelihood of HbS polymerization under normoxic conditions. Research has shown that reducing 2,3-DPG levels can delay HbS polymerization, as evidenced by experiments where reducing 2,3-DPG concentration by 50% led to a significant reduction in nucleation rates, thereby delaying the onset of sickling. Clinically, modulating 2,3-DPG levels could be a therapeutic strategy to reduce sickling. For instance, inhibitors of 2,3-DPG synthesis are being explored as potential treatments for SCA, as lowering 2,3-DPG concentrations in RBCs may enhance hemoglobin’s affinity for oxygen, preventing deoxygenation, and subsequent polymerization^[22]^.

## Inositol hexaphosphate (IHP)

IHP, similar to 2,3-DPG, affects the oxygen affinity of hemoglobin, but it binds even more strongly to deoxygenated HbS. Studies have shown that IHP acts as a potent modulator of HbS polymerization by stabilizing the deoxygenated state of HbS, making it an important factor in promoting nucleation. When IHP is present at high concentrations, it dramatically increases nucleation rates by enhancing the hydrophobic interactions between HbS molecules in the deoxygenated state. Experimental models have demonstrated that the addition of IHP to RBCs in vitro can accelerate nucleation by as much as 70%, highlighting its role as a key factor in promoting sickling. The clinical significance of IHP lies in its potential as a therapeutic target. Reducing the levels of IHP in RBCs could prevent the stabilization of deoxygenated HbS, thereby reducing nucleation rates and delaying the onset of polymerization. Although no IHP-targeting therapies are currently available, ongoing research aims to develop agents that can modulate IHP activity and mitigate its effects on HbS polymerization^[23]^.

## Linking mechanistic details to clinical manifestations

The mechanistic insights into nucleation provide a direct link to the clinical manifestations of SCA. The rapid onset of nucleation under conditions of high HbS concentration, low pH, and low oxygen tension explains the acute nature of VOCs, which are often triggered by stress, dehydration, or infection. The faster nucleation rates under these conditions lead to more frequent and severe sickling episodes, resulting in pain, tissue ischemia, and organ damage. Additionally, the chronic elevation of 2,3-DPG levels in patients with SCA contributes to the persistent risk of sickling, even in the absence of overt hypoxia. The relationship between nucleation and clinical outcomes is also evident in longterm complications of SCA. Repeated episodes of sickling and vaso-occlusion lead to chronic hemolysis, anemia, and damage to organs such as the lungs, kidneys, and brain. For example, the increased nucleation rates in hypoxic conditions contribute to the development of acute chest syndrome, a life-threatening complication characterized by pulmonary vaso-occlusion and infarction. Similarly, the role of nucleation in promoting RBC sickling under conditions of metabolic acidosis explains the association between SCA and renal dysfunction, as the kidneys are particularly susceptible to damage from repeated ischemic episodes^[24,25]^.

## Implications for SCA management

One of the primary implications of nucleation mechanisms for SCA management lies in the identification of pharmacological targets for therapeutic intervention^[23]^. By targeting specific molecular pathways involved in nucleation, such as the interactions between HbS molecules or the modulation of allosteric effectors, researchers can develop small molecule inhibitors or biologics capable of disrupting nucleation events. These targeted therapies have the potential to prevent or reduce the formation of HbS polymers, thereby alleviating vaso-occlusive crises, hemolysis, and other complications associated with SCA. Moreover, advancements in gene therapy and genome editing technologies hold promise for correcting the underlying genetic defect responsible for abnormal HbS polymerization in SCA^[24]^. By targeting the β-globin gene or other genes involved in nucleation pathways, researchers can potentially restore normal hemoglobin function and prevent the formation of sickled RBCs. Clinical trials investigating gene-based therapies for SCA are currently underway, offering hope for a curative approach to disease management^[25-28]^.

In addition to targeted pharmacological and genetic interventions, the elucidation of nucleation mechanisms in SCA has implications for disease monitoring and prognostication. Biomarkers associated with nucleation kinetics, such as levels of soluble HbS aggregates or markers of hemolysis, may serve as useful indicators of disease severity and response to therapy^[29]^. By monitoring these biomarkers over time, clinicians can assess disease progression, adjust treatment strategies, and optimize patient care. Furthermore, understanding nucleation mechanisms provides insights into potential risk factors and triggers for vasoocclusive crises and other acute complications of SCA^[30-33]^. Environmental factors that promote deoxygenation, such as high altitude or physical exertion, may exacerbate nucleation kinetics and precipitate acute events. By identifying and mitigating these triggers, clinicians can help reduce the frequency and severity of crises and improve patient quality of life.

## Opportunities for innovative treatments

In the quest to mitigate the impact of sickle cell anemia (SCA), significant strides have been made in targeting the molecular mechanisms that drive hemoglobin S (HbS) polymerization and nucleation. Innovative treatment strategies focus on preventing HbS polymerization, altering red blood cell (RBC) physiology, or addressing the downstream effects of sickling. A deeper understanding of nucleation mechanisms has guided the development of these therapies, which hold promise for reducing vasoocclusive crises (VOCs), hemolysis, and long-term complications in SCA patients. Recent clinical trials have demonstrated the efficacy and safety of these therapies, underscoring their potential to revolutionize the management of SCA^[29]^.

## Voxelotor

Voxelotor, a small molecule, represents a breakthrough in SCA treatment by directly targeting HbS polymerization. Voxelotor binds to the N-terminal valine of the alpha-globin chain, stabilizing the oxygenated form of hemoglobin (the R-state) and increasing hemoglobin’s affinity for oxygen. This prevents deoxygenation and the subsequent polymerization of HbS, which is the key trigger for RBC sickling. By preventing HbS from adopting the deoxygenated T-state, voxelotor reduces the nucleation of HbS polymers, thereby preventing the cascade of events leading to sickle cell crises. Molecularly, voxelotor’s mechanism ensures that HbS remains in its oxygenated form even at lower oxygen tensions, delaying or preventing the onset of polymerization and the formation of sickled cells. Clinical trials, such as the HOPE trial, have shown that voxelotor significantly increases hemoglobin levels in SCA patients, reduces markers of hemolysis, and lowers the frequency of sickle cell crises. Patients treated with voxelotor showed a reduction in VOCs and fewer hospitalizations, indicating both efficacy and safety. Moreover, voxelotor has been shown to maintain oxygen saturation in patients, preventing the hypoxia-driven sickling process at its source^[30]^.

## CRISPR-based gene editing

Gene editing technologies like CRISPR-Cas9 have emerged as a revolutionary approachtotreating SCAat thegenetic level.These techniques target the root cause of the disease by either correcting the mutation responsible for HbS or by increasing the production of fetal hemoglobin (HbF), which inhibits HbS polymerization. CRISPR-based therapies work by introducing targeted DNA cuts at specificsites in thegenome,allowing foreither thecorrection of the HbS mutation or the reactivation of genes that induce HbF production. One of the most promising approaches involves targeting the BCL11A gene, which suppresses HbF production in adults. By knocking out BCL11A, CRISPR technology enables the reactivation of HbF, which interferes with HbS polymerization by reducing its concentration and hindering nucleation.

Recent clinical trials, such as those conducted by CRISPR Therapeutics and Vertex Pharmaceuticals, have demonstrated that patients treated with CRISPR-edited hematopoietic stem cells showed increased levels of HbF, significantly reducing the incidence of VOCs and eliminating the need for transfusions. These patients have also exhibited long-lasting therapeutic effects, with sustained HbF production over time, suggesting a potential one-time curative treatment for SCA^[31,32]^.

## Lentiviral gene therapy

Another innovative approach is lentiviral gene therapy, which aims to restore the production of functional hemoglobin in SCA patients. In this strategy, a lentiviral vector is used to deliver a modified beta-globin gene into the patient’s hematopoietic stem cells. This corrected gene expresses a form of hemoglobin that does not polymerize like HbS, thereby preventing sickling. Once these modified stem cells are infused back into the patient, they produce RBCs that contain functional hemoglobin, reducing the sickling process. Bluebird Bio’s lentiviral gene therapy, known as LentiGlobin, has shown remarkable success in clinical trials. In patients treated with LentiGlobin, over 50% of hemoglobin produced was nonsickling, which dramatically reduced VOCs and hemolysis. Follow-up data from the HGB-206 trial revealed that patients experienced sustained increases in total hemoglobin levels and showed a reduction in the clinical symptoms of SCA. This therapy offers a potentially curative option, with the longterm goal of eliminating the disease by producing normal RBCs throughout the patient’s life^[32,33]^.

## Hydroxyurea

Hydroxyurea, a well-established therapy for SCA, exerts its effect by increasing the production of HbF, which reduces the concentration of HbS and inhibits its polymerization. HbF interferes with HbS nucleation by diluting HbS concentration within RBCs, delaying polymerization and preventing sickling. In addition to promoting HbF synthesis, hydroxyurea also reduces the expression of adhesion molecules on RBCs and leukocytes, decreasing the likelihood of vaso-occlusion in blood vessels. Quantitative data from clinical trials have demonstrated that hydroxyurea increases HbF levels by 10–20%, significantly reducing the frequency of VOCs and hospitalizations in patients with SCA.Longterm studies have shown that hydroxyurea improves overall survival and decreases the incidence of severe complications, such as acute chest syndrome and stroke. Despite its widespread use and efficacy, hydroxyurea is not a curative treatment and is limited by variability in patient response and potential long-term side effects^[30]^.

## Allosteric modifiers of hemoglobin

Beyond voxelotor, several other allosteric modifiers of hemoglobin are being developed to prevent HbS polymerization. These compounds work by stabilizing the oxygenated form of hemoglobin or altering the allosteric properties of hemoglobin to prevent deoxygenation-induced nucleation. Agents such as GBT440 (previously developed by Global Blood Therapeutics) act similarly to voxelotor by binding to hemoglobin and maintaining it in the R-state, thereby reducing the rate of HbS polymerization. Recent studies have explored the efficacy of other allosteric modifiers that target different sites on the hemoglobin molecule, offering additional ways to modulate HbS polymerization. These agents, in early-phase clinical trials, have shown promise in reducing sickling rates and improving RBC deformability, which is critical for preventing vaso-occlusion and subsequent ischemic damage. The development of such allosteric modulators could provide an expanded therapeutic arsenal for managing SCA, particularly in patients who do not respond to existing treatments like hydroxyurea^[31,32]^.

## Antioxidant therapies

Oxidative stress plays a crucial role in the pathophysiology of SCA, assickledRBCsgeneratereactiveoxygenspecies(ROS)that exacerbate hemolysis and endothelial damage. Targeting oxidative stress with antioxidant therapies is an emerging strategy for reducing the complications of SCA. Antioxidants work by scavenging ROS and reducing the oxidative damage that sickled RBCs inflict on vascular endothelium and other cells. For example, glutamine, an amino acid with antioxidant properties, has been approved by the FDA for the treatment of SCA under the trade name Endari. Clinical trials have shown that glutamine reduces VOCs and hospitalizations by decreasing oxidative stress and improving RBC function. Additionally, research on novel antioxidants like N-acetylcysteine (NAC) hasshown potential for reducing hemolysis and improving RBC survival, further highlighting the role of oxidative stress in SCA pathology^[33]^.

## Targeting ion channels

Abnormal ion channel function in sickled RBCs contributes to cell dehydration and increased intracellular HbS concentration, both of which promote nucleation and sickling. Therapeutic agents that target these ion channels offer a novel approach to preventing RBC dehydration and reducing HbS polymerization. For example, senicapoc, a Gardos channel inhibitor, works by blocking the potassium-calcium (KCa) channel in RBCs, preventing the efflux of potassium and water and maintaining cell volume. By preventing RBC dehydration, senicapoc reduces intracellular HbS concentration and delays nucleation. Although early clinical trials of senicapoc showed a reduction in hemolysis, the drug did not significantly reduce VOCs. However, ongoing research into other ion channel modulators may yield more effective treatments for preventing sickling by maintaining RBC hydration and deformability^[32]^.

## Clinical trials and future directions

Ongoing clinical trials for these innovative therapies continue to provide valuable insights into their efficacy and safety. For example, voxelotor’s HOPE trial demonstrated sustained improvements in hemoglobin levels and reductions in hemolysis markers, while CRISPR-based gene therapy trials have shown promising results in increasing HbF levels and reducing disease severity. As gene editing and gene therapy approaches evolve, their potential for providing a one-time cure for SCA grows increasingly tangible. Looking ahead, combination therapies that target multiple aspects of SCA pathophysiology—such as combining hydroxyurea with voxelotor or antioxidants—may offer synergistic benefits. Additionally, further researchinto thegenetic and molecular mechanisms underlying nucleation could lead to the development of even more targeted treatments. With advances in personalized medicine, it may soon be possible to tailor treatments to the specific genetic and biochemical profiles of individual patients, optimizing therapeutic outcomes, and reducing the global burden of SCA^[33]^.

## Current limitations of proposed therapeutic approaches and potential challenges in clinical implementation

While recent advances in sickle cell anemia (SCA) treatment offer promising avenues for reducing disease severity and improving patient outcomes, several limitations and challenges remain. These hurdles span from the molecular mechanisms of the therapies to broader concerns such as accessibility, cost, longterm safety, and patient variability in treatment response. Understanding these limitations is crucial for addressing the gaps in SCA management and for developing more effective, universally accessible therapeutic solutions.

## Voxelotor: limited long-term efficacy and variable patient response

Although voxelotor has demonstrated substantial efficacy in reducing HbS polymerization and preventing vaso-occlusive crises (VOCs), its long-term impact on disease progression is still being investigated. One limitation is the reliance on increasing oxygen affinity, which may, in some cases, lead to reduced tissue oxygen delivery despite higher hemoglobin levels. This paradox could potentially result in long-term complications, such as exacerbation of tissue hypoxia, particularly in patients with more severe forms of SCA. Moreover, not all patients respond equally to voxelotor. Factors such as genetic heterogeneity, variations in hemoglobin levels, and differing oxygen environments may influence individual responses to the drug. Additionally, voxelotor’s high cost poses a significant barrier, especially in low-resource settings where the burden of SCA is highest. Ensuring equitable access to this therapy remains a considerable challenge, as healthcare systems in many regions, particularly in sub-Saharan Africa, may lack the infrastructure to deliver voxelotor on a large scale^[34,35]^.

## CRISPR-based gene editing: ethical, safety, and access challenges

CRISPR-based gene editing holds immense potential for curing SCA at the genetic level. However, its implementation is fraught with ethical concerns, safety risks, and logistical challenges. The manipulation of human genomes raises complex ethical issues, particularly regarding the potential for unintended off-target effects or germline modifications, which could have heritable consequences. Although CRISPR technology has improved in terms of precision, the risk of introducing harmful mutations remains a significant limitation. Safety concerns also extend to the potential for immune responses to gene-edited cells, as well as the risk of clonal expansion of edited cells leading to malignancies. Long-term follow-up of patients undergoing CRISPR therapy is required to monitor these risks, but the technology is still too new to have established a complete safety profile over decades. Furthermore, the high costs of gene-editing procedures, combined with the need for sophisticated healthcare infrastructure, limit access to CRISPR-based therapies, particularly in regions where SCA is most prevalent. This disparity could exacerbate global health inequities, with wealthier nations gaining access to curative treatments while low-income regions struggle with outdated care options^[36,37]^.

## Lentiviral gene therapy: technical and financial constraints

Lentiviral gene therapy, like CRISPR, offers a promising approach to curing SCA by introducing a corrected hemoglobin gene. However, several technical and financial barriers impede its widespread use. The production and delivery of lentiviral vectors are technically complex, requiring sophisticated facilities for both vector manufacture and stem cell transplantation. This limits the scalability of lentiviral gene therapy, particularly in regions with under developed healthcare systems. In addition to logistical challenges, the cost of lentiviral gene therapy remains prohibitively high, making it inaccessible to most patients worldwide. Moreover, the therapy requires conditioning regimens (such as chemotherapy) to clear the patient’s existing hematopoietic stem cells, a process that comes with its own risks, including infection, organ toxicity, and infertility. Although early trials have shown promising results, the long-term safety of lentiviral gene therapy, particularly in children, remains unknown. There is also a concern about the durability of gene therapy effects and whether patients will require retreatment later in life^[38,39]^.

## Hydroxyurea: suboptimal responses and long-term side effects

Hydroxyurea has been a cornerstone of SCA management for decades, primarily due to its ability to increase fetal hemoglobin (HbF) levels and reduce VOCs. However, it has limitations related to patient variability in response. Some patients do not experience significant increases in HbF, leading to suboptimal clinical outcomes. The reasons for this variability are not fully understood but may involve genetic differences in HbF production pathways or variations in drug metabolism. Additionally, hydroxyurea is associated with several long-term side effects, including myelosuppression, which can increase the risk of infections. There are also concerns about its potential to cause reproductive toxicity and malignancy, particularly in patients who require life-long therapy. While hydroxyurea remains an effective treatment for many patients, its non-curative nature and side effect profile highlight the need for more advanced therapies that offer durable solutions with fewer risks^[40]^.

## Allosteric modifiers and antioxidants: limited data and accessibility issues

While allosteric modifiers of hemoglobin and antioxidant therapies such as glutamine have shown promise in reducing HbS polymerization and oxidative stress, their long-term efficacy and safety are still under investigation. For example, agents like voxelotor or GBT440 primarily target HbS polymerization, but their impact on the downstream complications of SCA, such as chronic organ damage, remains unclear. There is also limited data on the use of these agents in pediatric populations, which represent a significant portion of the global SCA burden. Moreover, antioxidant therapies like glutamine face challenges in terms of accessibility and affordability. While glutamine is now FDA-approved, its high cost and the need for regular administration may limit its use in resourcepoor settings. Further researchis neededto determine whether these therapies can be combined with other treatments to improve efficacy and whether they offer long-term protection against the systemic complications of SCA^[41]^.

## Targeting ion channels: early-stage development and efficacy concerns

Ion channel modulators, such as senicapoc, represent a novel approach to preventing RBC dehydration and reducing HbS concentration. However, early trials with senicapoc have shown mixed results. Although the drug reduces hemolysis and RBC dehydration, it has not consistently reduced VOC frequency in clinical trials, raising questions about its overall efficacy in preventing the most severe complications of SCA. Additionally, ion channel modulators are still in the early stages of development, and more research is needed to optimize their use. There are concerns about potential off-target effects, as ion channels are involved in various physiological processes, and blocking these channels could result in unintended side effects. As such, ion channel modulators may need to be used in combination with other therapies to achieve clinically meaningful outcomes in SCA^[42]^.

## Accessibility and health system barriers

Beyond the molecular and clinical limitations of these therapies, broader systemic barriers also challenge the global implementation of innovative treatments for SCA. In many low-income countries and middle-income countries (LMICs), where SCA prevalence is highest, healthcare systems are under-resourced and ill-equipped to provide advanced therapies like gene editing or even regular hydroxyurea therapy. Diagnostic infrastructure is often lacking, leading to delays in diagnosis and initiation of treatment. Even in high-income countries, the costs of advanced therapies like CRISPR-based gene editing or lentiviral gene therapy are prohibitive for many patients. These therapies often require specialized care centers, long-term follow-up, and supportive care, further limiting their accessibility. Addressing these barriers will require coordinated efforts between governments, global health organizations, and pharmaceutical companies to ensure equitable access to life-saving treatments^[43]^.

## Personalized treatments and the reduction of vaso-occlusive crises: emphasizing precision medicine and biomarker identification

As sickle cell anemia (SCA) treatment evolves, there is growing recognition that personalized approaches can significantly improve outcomes, particularly in reducing the frequency and severity of vaso-occlusive crises (VOCs). VOCs are the hallmark of SCA, resulting from sickled red blood cells (RBCs) obstructing microvasculature, causing pain, tissue ischemia, and long-term organ damage. Traditional treatments have often taken a generalized approach, but precision medicine offers a more individualized strategy, tailoring treatment to the unique molecularand genetic profiles of each patient. Central to this approach is the identification of specific biomarkers that can predict disease severity, VOC risk, and therapeutic response, enabling more targeted and effective interventions^[39]^.

## The role of precision medicine in SCA management

Precision medicine in SCA aims to address the variability in disease presentation and treatment response by considering individual differences in genetics, environment, and lifestyle. For instance, patients with SCA exhibit varying degrees of HbF production, red blood cell membrane fragility, and oxidative stress, all of which influence the likelihood and frequency of VOCs. By leveraging genetic and molecular information, clinicians can better predict which patients are at higher risk for severe VOCs and tailor therapies accordingly. This reduces the risk of over-treatment or under-treatment, which can occur when a one-size-fits-all approach is applied. For example, patients with higher levels of HbF (fetal hemoglobin) tend to have milder disease courses and fewer VOCs. Hydroxyurea, a drug that induces HbF production, has long been used to reduce VOC frequency, but notallpatients respondequally. Precision medicine allows for the identification of patients who are more likely to benefit from hydroxyurea therapy based on their genetic profile, optimizing its use. Moreover, precision medicine can help identify alternative therapeutic targets for patients who do not respond well to hydroxyurea, providing a more personalized approach to managing VOCs^[40]^.

## Biomarker identification

Biomarker identification is fundamental to the development of personalized treatments for SCA. Biomarkers are measurable indicators of biological processes, disease states, or treatment responses, and they can provide valuable insights into the mechanisms driving VOCs. Several promising biomarkers have been identified in SCA that correlate with VOC frequency and severity, offering opportunities for more targeted interventions^[41]^.

1. Inflammatory markers: VOCs are often preceded by systemic inflammation, with elevated levels of cytokines such as TNF-α, IL-1β, and IL-6. These markers not only indicate the presence of inflammation but also contribute to the activation of endothelial cells and increased adhesion of sickled RBCs to the vasculature. Monitoring these inflammatory biomarkers can help predict impending VOCs, allowing for early intervention with anti-inflammatory therapies or other preventive measures.
2. Endothelial activation: Endothelial dysfunction plays a crucialrole in VOCs, as the adhesion of sickled RBCs to the endothelium triggers vaso-occlusion. Biomarkers such as soluble VCAM-1, ICAM-1, and E-selectin are indicators of endothelial activation and can serve as predictors of VOCs. Identifying patients with higher levels of these biomarkers can guide the use of treatments that target the endothelium, such as selectin inhibitors (e.g. crizanlizumab), which have been shown to reduce VOC frequency by inhibiting RBC-endothelial adhesion.
3. Oxidative stress markers: SCA patients experience increasedoxidative stress due to chronic hemolysis and inflammation, contributing to VOCs. Biomarkers such as malondialdehyde (MDA) and glutathione (GSH) levels reflect the degree of oxidative stress and can guide antioxidant therapies. For instance, glutamine supplementation has been shown to reduce oxidative stress and VOCs in some patients, and precision medicine could help identify those most likely to benefit from such therapies based on their oxidative stress profiles.
4. Red blood cell dehydration and ion channel activity: The dehydration of sickled RBCs exacerbates polymerization and sickling. Ion channel modulators, such as senicapoc, target RBC dehydration by blocking potassium channels (Gardos channels). By measuring biomarkers related to RBC hydration status and ion channel activity, clinicians can identify patients who may benefit from these treatments, ensuring a more precise therapeutic approach.
5. Genetic modifiers: Genetic variations in HbF production, nitric oxide metabolism, and adhesion molecule expression have been linked to differences in VOC frequency and disease severity. For example, polymorphisms in the BCL11A and MYB genes influence HbF levels, which can affect the clinical course of SCA. Precision medicine aims to integrate genetic screening into routine clinical practice to identify these modifiers, allowing for more personalized treatment plans, such as optimizing hydroxyurea dosing or selecting patients for gene therapy.

## Personalized therapies

Recent advances in personalized therapies for SCA have focused on targeting specific molecular pathways involved in VOCs. These therapies are designed to interfere with the processes that lead to vaso-occlusion at the cellular and molecular levels, providing more effective and individualized treatment options^[42]^.

1. Crizanlizumab: Crizanlizumab, a monoclonal antibody targeting P-selectin, prevents the adhesion of sickled RBCs to the endothelium, a critical step in the initiation of VOCs. By blocking this adhesion, crizanlizumab reduces the frequency of VOCs in patients with moderate to severe SCA. The use of biomarkers for endothelial activation could further enhance its efficacy by identifying patients most likely to benefit from this treatment.
2. Voxelotor: Voxelotor, a hemoglobin modifier, works by increasing the oxygen affinity of HbS, preventing its polymerization and subsequent sickling. While voxelotor has been shown to reduce VOCs and hemolysis, its efficacy may vary based on the patient’s HbS concentration and oxygen environment. Precision medicine can help optimize voxelotor therapy by identifying patients with specific HbS polymerization rates and oxygen delivery challenges, allowing for more targeted treatment regimens.
3. Gene therapy: Gene-editing technologies such as CRISPR-Cas9 and lentiviral gene therapy offer the potential for a personalized cure by directly correcting the genetic mutation responsible for SCA. Patients with specific genetic profiles, such as those with certain HbF-promoting polymorphisms, may be ideal candidates for gene therapy. Biomarker identification and genetic screening can help tailor gene-editing approaches to individual patients, ensuring the highest likelihood of success while minimizing risks.

## Limitations of current approaches and challenges for clinical implementation

Despite the promising advances in personalized treatments for sickle cell anemia (SCA), there are several limitations and challenges that hinder the widespread adoption and effectiveness of these therapies. These challenges range from the inherent complexity of the disease, the variability in patient response, and the high costs of new therapies, to issues related to accessibility, infrastructure, and the need for further clinical validation.

1. Heterogeneity in disease presentation and response to treatment

One of the most significant challenges in SCA treatment is the heterogeneity of thedisease. SCA patients exhibit a wide range of clinical symptoms, from mild anemia to frequent and severe vaso-occlusive crises (VOCs). This variability is influenced by several factors, including genetic modifiers (e.g. variations in HbF levels), environmental factors (e.g. exposure to oxidative stress), and comorbidities. As a result, treatments that are effective for one group of patients may not work as well for others. For example, hydroxyurea, which induces fetal hemoglobin (HbF) production, has proven highly effective in some patients. However, a significant portion of patients either do not respond adequately or experience severe side effects. The same variability is seen with newer treatments like crizanlizumab and voxelotor, where responses can be inconsistent depending on individual patient characteristics. This variability complicates the development of standardized treatment protocols and highlights the need for further research into personalized treatment approaches based on precise genetic and molecular profiling^[30]^.

2. Biomarker identification and validation

While biomarker-driven precision medicine holds great potential, the identification and validation of reliable biomarkers remain a major limitation. Biomarkers such as inflammatory cytokines, endothelial adhesion molecules, and oxidative stress markers are still in the early stages of research, and many have not yet been fully validated for routine clinical use. The development of robust, reproducible biomarkers is essential for guiding personalized therapies, but the process of discovering, validating, and integrating these biomarkers into clinical practice is time-consuming and costly. Furthermore, many biomarkers may be influenced by multiple factors unrelated to SCA, such as infection, inflammation, or coexisting diseases, which could lead to inaccurate assessments of disease activity or treatment response. Developing biomarkers that are specific to SCA pathophysiology remains a significant challenge and is critical for the success of precision medicine approaches in this population^[39]^.

3. Cost and accessibility of advanced therapies

One of the primary barriers to implementing innovative treatments like crizanlizumab, voxelotor, and gene therapy is their cost. These therapies are often prohibitively expensive, limiting access for many patients, especially in resourcelimited settings such as sub-Saharan Africa, where SCA prevalence is highest. For example, crizanlizumab and voxelotor are estimated to cost tens of thousands of dollars per year per patient, making them inaccessible to most people in low-income countries and middle-income countries (LMICs). The infrastructure required to deliver gene therapy, including specialized facilities and highly trained personnel, further exacerbates these accessibility issues. Even in high-income countries, the cost of these therapies poses a significant burden on healthcare systems and patients. Insurance coverage and reimbursement for these advanced therapies can be challenging, especially for treatments that are still considered experimental or have not yet been widely adopted^[40]^.

4. Complexity and risks of gene therapy

Gene therapy represents one of the most promising potential cures for SCA, but its implementation in clinical practice faces several challenges. Although early trials using gene-editing technologies like CRISPR-Cas9 or lentiviral vectors have shown promise, the long-term safety and efficacy of these treatments remain uncertain. Concerns about off-target effects, immunogenicity, and the durability of gene correction have not been fully resolved. Additionally, the process of autologous stem cell transplantation, which is often required in gene therapy, carries significant risks, including graftversus-host disease (GVHD), infections, and other complications associated with high-dose chemotherapy. Moreover, the cost and complexity of gene therapy present substantial barriers to its widespread use. Currently, gene therapy is only available at a few specialized centers, and the procedures are labor-intensive and resource-demanding, making it difficult to scale for the broader SCA population. Efforts to reduce costs, improve safety, and streamline the production and delivery of gene therapies are needed to make them viable on a larger scale^[41]^.

5. Infrastructure and healthcare disparities

The successful implementation of personalized treatments for SCA, particularly in resource-limited settings, requires significant improvements in healthcare infrastructure. Precision medicine approaches rely on advanced diagnostic tools, genetic screening, and molecular profiling, which are not readilyavailable inmanyregions,particularly inAfrica, where SCA is most prevalent. A lack of access to modern laboratories, trained personnel, and the necessary technologies for biomarker testing or genetic therapies limits the ability of healthcare providers to offer personalized treatments. Healthcare disparities also play a role in limiting access to innovative treatments. In high-income countries, African American populations, who are disproportionately affected by SCA, often face barriers to healthcare, including limited access to specialists, lower rates of insurance coverage, and systemic healthcare inequities. These disparities further exacerbate the challenge of implementing personalized medicine approaches and ensuring that all patients receive the most effective treatments^[42]^.

6. Regulatory and ethical considerations

Innovative treatments such as gene therapy and biomarkerdriven therapies face regulatory hurdles that can delay their implementation in clinical practice. Regulatory agencies, such as the U.S. Food and Drug Administration (FDA) and the European Medicines Agency (EMA), require extensive clinical trial data to demonstrate the safety, efficacy, and long-term benefits of new treatments. The rigorous nature of these trials, while essential for patient safety, can slow down the availability of promising therapies. Additionally, ethical concerns surrounding gene editing, particularly germline editing, have sparked global debates. While current SCA gene therapies focus on somatic cell editing, which does not affect future generations, ongoing discussions about the ethical implications of genetic manipulation could influence the pace of research and clinical application^[43]^.

7. Limited long-term data

Many of the innovative therapies for SCA are relatively new, and long-term data on their safety and efficacy are still limited. For instance, voxelotor and crizanlizumab have only been available for a few years, and while early results are promising, questions remain about their long-term impact on disease progression, organ damage, and overall survival. Similarly, gene therapy is in its early stages, and while initial trials show high efficacy, long-term outcomes, such as the durability of gene correction and potential late-onset side effects, remain unknown. Without comprehensive long-term data, clinicians may be hesitant to adopt these treatments widely, and patients may be reluctant to opt for them without assurances of sustained benefit and safety. Continued research and long-term follow-up studies are crucial to addressing these concerns^[43]^.

## Future directions

Despite significant progress in elucidating nucleation mechanisms and developing targeted therapies for sickle cell anemia (SCA), several avenues for future research and clinical translation remain. These future directions aim to further enhance our understanding of nucleation processes and improve patient outcomes through innovative approaches to disease management.

1. Precision medicine approaches: Advances in genomic medicine and personalized therapeutics offer opportunities totailor treatment strategies to individual patients based on their genetic profile, disease phenotype, and response to therapy^[34]^. Integrating genomic data with clinical parameters may facilitate the identification of patient-specific biomarkers and predictive models for treatment response, enabling more precise and effective interventions^[44]^.
2. Novel therapeutic targets: Continued exploration of nucleation pathways and associated molecular targets may lead to the discovery of novel therapeutic agents with enhanced specificity and efficacy. High-throughput screening assays, computational modeling, and structural biology approaches can aid in the identification and optimization of small molecule inhibitors, biologics, and gene-based therapies targeting nucleation mechanisms^[45]^.
3. Combination therapies: Given the multifactorial nature ofSCA pathophysiology, combination therapies targeting multiple aspects of the disease process, including nucleation, hemolysis, inflammation, and endothelial dysfunction, hold promise for synergistic therapeutic effects and improved clinical outcomes. Clinical trials evaluating combination regimens comprising nucleation inhibitors, disease-modifying agents, and supportive care measures are warranted to assess their safety and efficacy^[46]^.
4. Gene editing technologies: Advances in genome editing technologies, such as CRISPR-Cas9, offer potential avenues for correcting the underlying genetic mutation responsible for abnormal hemoglobin polymerization in SCA^[47]^. Preclinical studies have demonstrated the feasibility of gene editing approaches for restoring normal hemoglobin function and preventing sickling in animal models. Translating these findings into clinical applications requires further optimization of delivery systems, off-target effects, and long-term safety considerations.
5. Biomarker development: The identification of reliable biomarkers associated with nucleation kinetics, disease severity, and treatment response is essential for disease monitoring, prognostication, and therapeutic development in SCA^[38]^. Longitudinal studies evaluating the utility of circulating biomarkers, imaging modalities, and novel assays for assessing nucleation activity and disease progression are needed to validate their clinical utility and inform decision-making in patient care.
6. Health equity and access to care: Addressing disparities inaccess to care, resources, and research opportunities for individuals with SCA is critical for ensuring equitable outcomes and reducing health disparities^[39]^. Collaborative efforts between researchers, healthcare providers, policymakers, and community stakeholders are essential for implementing culturally competent care models, expanding access to innovative therapies, and advocating for policies that prioritize SCA research and education.

## Conclusion

The elucidation of nucleation mechanisms in sickle cell anemia (SCA) represents a critical milestone in our understanding of this complex genetic disorder. Nucleation, the initial step in the polymerization of hemoglobin S (HbS) molecules, plays a central role in SCA pathophysiology, influencing disease severity, clinical manifestations, and treatment outcomes. By unraveling the molecular intricacies of nucleation, researchers have identified potential targets for therapeutic intervention and developed innovative strategies aimed at mitigating disease burden and improving patient quality of life. The implications of nucleation mechanisms extend beyond basic science research to encompass clinical practice, disease monitoring, and therapeutic development in SCA. Targeted pharmacological agents, gene editing technologies, and precision medicine approaches hold promise for transforming the landscape of SCA management, offering hope for more effective treatments and ultimately a cure for this debilitating disorder. Furthermore, the identification of reliable biomarkers and the promotion of health equity are essential for ensuring equitable access to care and optimizing patient outcomes across diverse populations affected by SCA.
